# Investigating the Therapeutic Potential of the Ketogenic Diet in Modulating Neurodegenerative Pathophysiology: An Interdisciplinary Approach

**DOI:** 10.3390/nu17071268

**Published:** 2025-04-04

**Authors:** Iqra Shabbir, Keying Liu, Bakhtawar Riaz, Muhammad Farhan Rahim, Saiyi Zhong, Jude Juventus Aweya, Kit-Leong Cheong

**Affiliations:** 1 College of Food Science and Technology, Guangdong Ocean University, Zhanjiang 524088, China; iqrashabbir@stu.gdou.edu.cn (I.S.); 17852980891@stu.gdou.edu.cn (K.L.); bakhtawarriaz2000@gmail.com (B.R.); zhongsy@gdou.edu.cn (S.Z.); 2 Department of Clinical Studies, Arid Agriculture University, Rawalpindi 43600, Pakistan; muhammadfarhanr@uaar.edu.pk; 3 Department of Food and Human Nutritional Sciences, University of Manitoba, Winnipeg, MB R3T 2N2, Canada; 4 The Canadian Centre for Agri-Food Research in Health and Medicine, St Boniface Hospital Albrechtsen Research Centre, Winnipeg, MB R2H 2A6, Canada

**Keywords:** ketogenic diet, neurodegenerative diseases, Alzheimer’s disease, Parkinson’s disease, beta-hydroxybutyrate, cognitive function, mitochondrial function, neuroprotection

## Abstract

The ketogenic diet (KD) is a dietary intervention comprising a high-fat, low-carbohydrate, and moderate-protein intake designed to induce a metabolic state known as ketosis, whereby ketone bodies are produced as an alternative source of energy. Initially established as a treatment for intractable epilepsy, the KD has subsequently gained significant attention for its potential to manage neurodegenerative diseases, including Alzheimer’s, Parkinson’s, and Huntington’s disease. Ketone bodies, such as beta-hydroxybutyrate (BHB), have been demonstrated to possess neuroprotective properties. The increasing prevalence of neurodegenerative diseases, such as Alzheimer’s, Parkinson’s, and Huntington’s disease, poses a significant public health challenge worldwide. With neurological disorders being the second-leading cause of death globally, the need for effective therapeutic interventions has never been more urgent. Recent evidence suggests that dietary interventions, particularly the ketogenic diet, offer promising potential in mitigating the progression of these diseases by influencing metabolic processes and providing neuroprotective benefits. The ketogenic diet, characterized by high-fat and low-carbohydrate intake, induces ketosis, leading to the production of ketone bodies like beta-hydroxybutyrate, which enhance mitochondrial efficiency, reduce oxidative stress, and modulate inflammatory pathways—mechanisms critical in neurodegenerative pathophysiology. This review explores the role of the ketogenic diet in managing neurological conditions, examining its mechanisms of action, historical context, and therapeutic efficacy. The paper also discusses emerging evidence linking the ketogenic diet to improved cognitive function, reduced motor symptoms, and enhanced mitochondrial activity in patients with neurodegenerative disorders. Additionally, the review highlights the need for further research to refine the therapeutic applications of the ketogenic diet, investigate its impact on various neurodegenerative diseases, and better understand its potential long-term effects. This study underscores the importance of nutrition as a vital aspect of the treatment strategy for neurological diseases, advocating for continued exploration of dietary interventions to improve brain health and function.

## 1. Introduction

There is growing global concern about the increasing prevalence of neurological disorders, particularly neurodegenerative diseases, which represent a significant public health burden. A considerable proportion of the global population is becoming increasingly concerned about the rising prevalence of neurological disorders, particularly neurodegenerative diseases. The findings of the Global Burden of Disease (GBD) study serve to reinforce the gravity of this issue, underscoring its pivotal implications for global health. This issue becomes even more concerning when considering the findings of the Global Burden of Disease (GBD) study, which highlights the substantial burden of neurological disorders on public health [1]. Neurological disorders are responsible for approximately 9 million deaths annually, representing 16.5% of global fatalities, the second-leading cause of death after cardiovascular diseases. Moreover, they are the primary cause of disability, resulting in a notable increase in the disability-adjusted life years (DALY) score. This metric quantifies the number of years lost due to premature mortality or living with a disability, thereby highlighting a burden that surpasses even that posed by the ongoing global pandemic of the novel coronavirus (2019-nCoV). However, the implications of neurological disorders extend far beyond those observed during the pandemic, underscoring the urgency of addressing this pressing health crisis in a comprehensive manner [2]. This trend may indicate an impending surge in neurological diseases, which would have significant implications for public health. Further investigation is therefore warranted. The emerging evidence suggests that post-viral neurological sequelae, potentially triggered by SARS-CoV-2 infection, could exacerbate the prevalence of neurodegenerative disorders in the coming years. It has been demonstrated that the systemic inflammation and immune dysregulation induced by SARS-CoV-2 may accelerate pathological processes such as amyloid beta aggregation and tau phosphorylation, which are hallmarks of Alzheimer’s disease. Recent research findings indicate that individuals who have tested positive for the novel coronavirus (SARS-CoV-2) are no more susceptible to developing neurological disorders than those who have contracted other respiratory tract infections. There have been reports of potential neurological complications, including neurodegenerative disorders, following the onset of SARS-CoV-2 infection [3]. An ischemic stroke represents an exception to this trend, as it has been demonstrated to increase the risk of developing epilepsy by a factor of approximately two [4]. The rising prevalence of neurological disorders, particularly those occurring in the wake of the ongoing pandemic, underscores the necessity for sophisticated investigative techniques to elucidate the underlying mechanisms. It has been demonstrated that neuroinflammation and immune dysregulation resulting from infection with the SARS-CoV-2 virus may increase the risk of developing conditions such as Alzheimer’s disease and epilepsy. The utilization of advanced research tools, such as molecular imaging and biomarkers, is of paramount importance for the exploration of these intricate processes and the development of targeted treatments. Dietary interventions represent a crucial aspect of the multifaceted treatment approach to neurological diseases. Nutrition plays a pivotal role in the management of neurological disorders, exerting a direct influence on metabolic processes and neuroprotection. Among the various dietary interventions, the ketogenic diet has emerged as a prominent strategy, supported by nearly a century of research and clinical application. Evidence indicates that the ketogenic diet facilitates neuroprotective mechanisms, including the reduction of oxidative stress, inflammation, and mitochondrial dysfunction [5]. This study examines the potential therapeutic benefits of the ketogenic diet for the treatment of neurodegenerative disorders, including Parkinson’s, Alzheimer’s, and Huntington’s diseases. This text examines the potential mechanisms of action of the ketogenic diet, including the production of ketone molecules such as beta-hydroxybutyrate. It has been demonstrated that ketone bodies, in particular beta-hydroxybutyrate, enhance mitochondrial efficiency and neuronal energy metabolism, thereby attenuating neurodegeneration [6]. The evidence that the ketogenic diet can assist in enhancing brain function and lessening the outward symptoms of various diseases is also examined in this study. It also emphasizes the critical need for further research to refine the therapeutic efficacy of the ketogenic diet and gain a comprehensive understanding of its impact on neurodegenerative diseases. This diet includes a high fat intake and a low carbohydrate intake, leading the body to enter a state of ketosis, where it manufactures ketone bodies as an alternate energy source. By lowering oxidative stress, inflammations, and cell death, common characteristics of neurodegenerative diseases, ketone bodies, especially beta-hydroxybutyrate, are projected to have neuroprotective effects. Animal and human studies further suggest that the ketogenic diet may improve cognitive performance, reduce movement symptoms, and boost mitochondrial activity in individuals with neurodegenerative diseases. For instance, recent trials have indicated significant improvements in cognitive scores among Alzheimer’s disease patients and reduced motor symptoms in Parkinson’s disease patients adhering to ketogenic interventions [7].

## 2. A Detailed Exploration of How Ketosis, Metabolic Shifts, and Hormonal Regulation Contribute to Therapeutic Effects

### 2.1. Induction of Ketosis and the Shift in Energy Sources: Mechanisms and Implications

The ketogenic diet induces a state of ketosis, whereby the body increases the production of ketone bodies (acetoacetate, acetone, and beta-hydroxybutyrate). This metabolic state is brought about by a substantial reduction in carbohydrate intake, which forces the body to derive energy primarily from fats. In this process, fatty acids are metabolized in the liver to produce ketone bodies, which serve as an alternative energy source for cells, particularly in the brain. Furthermore, fasting or carefully adhering to a low-calorie diet (which does not necessarily need to be high in fat) may also result in a state of ketosis. However, it is essential to distinguish this state from nutritional ketosis, which is specifically achieved through a high-fat, low-carbohydrate ketogenic diet [8].

### 2.2. The Composition and Mechanism of the Ketogenic Diet

Building upon insights from the existing literature, the ketogenic diet can be precisely characterized by its macronutrient composition, which is tailored to induce a metabolic state of ketosis. This entails the consumption of 1.0, 1.2, or 1.7 g of protein per kilogram of body weight, which accounts for approximately 20% of the total caloric intake. Furthermore, carbohydrate intake is limited to a maximum of 50 g per day, typically accounting for 5–10% of the total energy intake. Meanwhile, fats constitute the majority of the diet’s total energy content, typically comprising 60–90% (most commonly 70–75%) of the diet’s total energy. The objective of the ketogenic diet is to replicate the physiological effects of fasting while avoiding the adverse effects associated with prolonged starvation. Both fasting and adherence to a ketogenic diet result in an increase in the breakdown of fatty acids and the utilization of ketone bodies (beta-hydroxybutyrate, acetoacetate, and acetone) as the primary energy source. This distinctive metabolic adaptation distinguishes the ketogenic diet from other dietary regimens, as it derives energy primarily from fats that have been converted into ketone bodies, rather than from glucose [9].

### 2.3. Investigation of the Neuroprotective Effects of Ketone Bodies

It has been demonstrated that ketone bodies, in particular beta-hydroxybutyrate (3HB), can enhance mitochondrial efficiency and neuronal energy metabolism. This process has been demonstrated to mitigate neurodegeneration and may promote neuroprotective effects by reducing oxidative stress and inflammation. Furthermore, ketone bodies act as signaling molecules that enhance mitochondrial function, reduce apoptosis, and modulate inflammatory pathways, which play a significant role in the pathogenesis of neurodegenerative diseases [10].

### 2.4. The Historical Context and Mechanism of Action of the Subject

The therapeutic concept behind the ketogenic diet (KD) originates from ancient observations that fasting could significantly alleviate seizures, especially in individuals with epilepsy. Historical records dating back to the fifth century BC suggest that Hippocrates advocated fasting as a natural remedy for seizures, while religious texts, such as the *Gospel of Saint Mark*, also reference healing through fasting and prayer. These early reports laid the groundwork for understanding the metabolic connection between nutrient intake and neurological function. This connection is artistically captured in Raphael’s painting *The Transfiguration*, which visually depicts the curative power of spiritual and physical fasting. Building upon these ancient practices, the early 20th century marked a pivotal moment in the development of dietary-based therapies for epilepsy. In 1921, Dr. Russell Wilder at the Mayo Clinic introduced the ketogenic diet as a scientifically structured alternative to fasting. His formulation aimed to replicate the beneficial metabolic effects of fasting—specifically, ketosis—without subjecting patients to the adverse effects of long-term caloric restriction. The original dietary prescription included 10–15 g of carbohydrates, 1 g of protein per kilogram of body weight, and the remaining energy from fats.

Since its inception, the KD has gained recognition as an effective treatment for drug-resistant epilepsy, particularly in pediatric populations. Although not considered a first-line therapy today, its efficacy in reducing seizure frequency and severity is well established through clinical and experimental research spanning nearly a century [11].

### 2.5. A Historical Overview of the Ketogenic Diet and Its Therapeutic Efficacy

**Figure 1:**
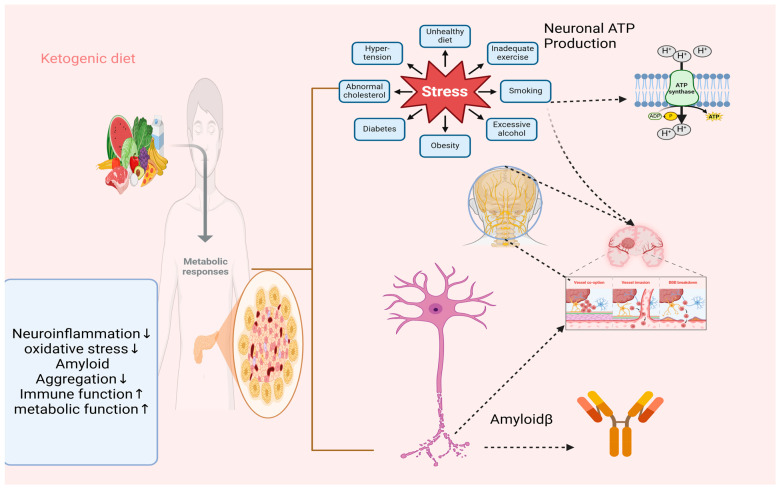
The proposed method by which the ketogenic diet may maintain brain health and enhance cognitive function.

While the initial application of the ketogenic diet focused on epilepsy, emerging evidence now supports its broader neuroprotective potential, particularly for neurodegenerative diseases such as Alzheimer’s, Parkinson’s, and Huntington’s disease. Mechanistic studies suggest that ketone bodies—especially beta-hydroxybutyrate—exert anti-inflammatory, antioxidant, and mitochondrial-stabilizing effects, making them promising therapeutic agents beyond epilepsy. As shown in Figure 1 the given diet’s established efficacy and metabolic foundations, it is scientifically rational to explore its therapeutic relevance in other neurological contexts [12]. Since its introduction, the ketogenic diet has been demonstrated to be an efficacious treatment for epilepsy, and continues to be a widely utilized alternative treatment for refractory epilepsy. However, it is not considered a standard treatment [13]. A wealth of research conducted over the past century has consistently validated the efficacy of the ketogenic diet in a multitude of clinical and experimental contexts. Although its initial application has been in managing epilepsy, an increasing body of evidence suggests that its neuroprotective mechanisms warrant further investigation for other neurological conditions, particularly neurodegenerative diseases. Given its historical efficacy in treating epilepsy, it is logical to investigate its potential role in mitigating the progression of neurodegenerative disorders (e.g., Alzheimer’s, Parkinson’s, and Huntington’s disease). It is imperative to conduct a comprehensive review of the current knowledge on epilepsy and other neurological illnesses to facilitate progress in research within this field, particularly given that almost a century has elapsed since its groundbreaking use.

### 2.6. The Production of Ketone Bodies and Metabolic Adaptation

When someone goes on a few days of fasting or drastically cuts the amount of carbs they eat every day to less than 20 g, their glucose stores are not enough to do two important things [14], normal fat oxidation by providing oxaloacetate in the Krebs cycle and [15] providing glucose to the central nervous system (CNS) [16].

In the case of glucose deprivation, oxaloacetate is required for the tricarboxylic acid cycle to function correctly, in a manner analogous to that described in point. Oxaloacetate is unable to accumulate within the mitochondrial matrix due to its inherent instability at physiological temperatures. Oxaloacetate is synthesized by ATP-dependent pyruvate carboxylase, which adds a carboxy group to pyruvic acid in the anaplerotic cycle [17].

### 2.7. Ketone Bodies and Their Role in Energy Metabolism Within the Central Nervous System (CNS)

**Figure 2:**
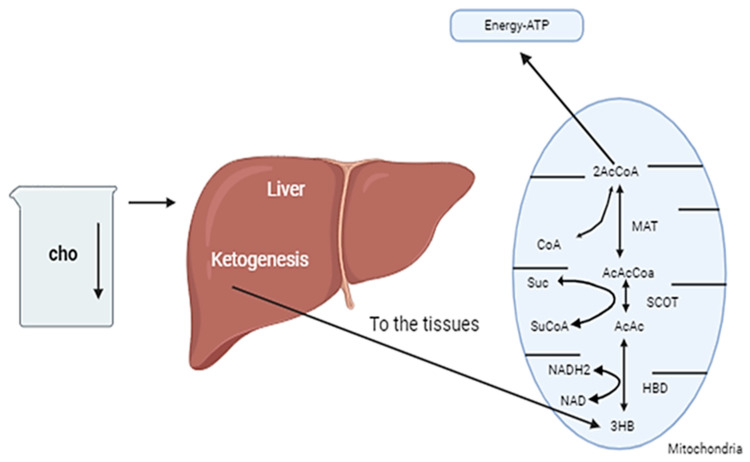
A reduction in carbohydrate intake can result in an elevation of the ketone bodies (KBs) produced by the liver. The liver is unable to utilize ketone bodies (KBs) due to the absence of the mitochondrial enzyme succinyl-CoA:3-ketoacid (alkaloid). The enzyme acetoacetate transferase (SCOT) facilitates the conversion of acetoacetate to acetoacetyl-CoA. Following this, succinyl-CoA:3-CoA transferase (SCOT) and methylacetoacetyl-CoA thiolase (MAT) facilitate the conversion of succinyl-CoA into acetyl-CoA, which allows for the entry of KBs into the citric acid cycle.

Fatty acids (FFAs) are unable to cross the blood–brain barrier, making glucose the dominant energy substrate for the brain. According to seminal investigations [18], a reduction in carbohydrate intake for a period of three to four days results in the central nervous system (CNS) utilizing alternative energy sources, namely ketone bodies (KBs). Ketone bodies represent an efficient alternative energy source during prolonged fasting or carbohydrate deprivation. They are synthesized through the process of ketogenesis, which is driven by an excess of acetyl-CoA and a concurrent reduction in oxaloacetic acid. This group comprises acetone, 3-hydroxybutyrate (3HB), and acetoacetate (AcAc: 18), all of which are ketone bodies. As shown in Figure 2 the majority of ketogenesis occurs within the mitochondrial matrix of the liver [19]. The liver is capable of synthesizing ketone bodies (KBs), yet lacks the requisite enzyme succinyl-CoA. 3-CoA transferase (SCOT) is essential for the conversion of acetoacetate into acetoacetyl-CoA, thereby preventing its utilization within the liver itself [20]. The most prevalent ketone in the bloodstream is 3-hydroxybutyrate, while the primary ketone produced in the liver is acetoacetate. Tissues such as the heart and skeletal muscle are capable of metabolizing small quantities of free acetoacetic acid, which is naturally produced in minute amounts. Large quantities of acetoacetic acid are produced, resulting in elevated levels that exceed the normal range. This is followed by conversion into the other two ketone bodies. As ketone bodies are excreted via the renal excretion system, they can be detected in both the blood (ketonemia) and urine (ketonuria). In conditions deemed normal, the concentration of ketone bodies (KBs) is less than 0.3 mmol/L, a figure that is considerably lower than that of glucose [21]. The CNS begins to utilize KBs as an alternative energy source when blood concentration levels reach approximately 4 mmol/L, which is in close proximity to the Km for the monocarboxylate transporter. The generation of energy by the body occurs through the conversion of ketone bodies (KBs) into acetoacetyl-CoA, a process initiated by the transformation of 3HB into AcAc [22]. It is intriguing to postulate that ketone bodies (KBs) may be capable of providing a greater quantity of adenosine triphosphate (ATP) than glucose. This is due to the fact that KBs have been observed to enhance mitochondrial efficiency in ATP production [23].

It is noteworthy that glycemia remains within the normal range despite a decline, as illustrated in Table 1. This effect is largely attributed to glucogenic amino acids and glycerol, which are derived from lipid catabolism. The highest concentration of ketones in the blood is 7–8 mmol/L during fasting or extremely low-calorie ketosis, also known as metabolic ketosis, with no alteration in the blood pH. In cases of untreated diabetic ketoacidosis, ketone body levels may exceed 25 mmol/L, as evidenced by the provided data, leading to a reduction in blood pH to below 7.3. In healthy adults, KB levels in the bloodstream typically remain below 8 mmol/L due to the brain and spinal cord’s efficient utilization of these molecules as an energy source, replacing glucose [24].

## 3. The Efficacy of the Ketogenic Diet in the Treatment of Neurodegenerative Diseases

### 3.1. Alzheimer’s Disease

**Figure 3:**
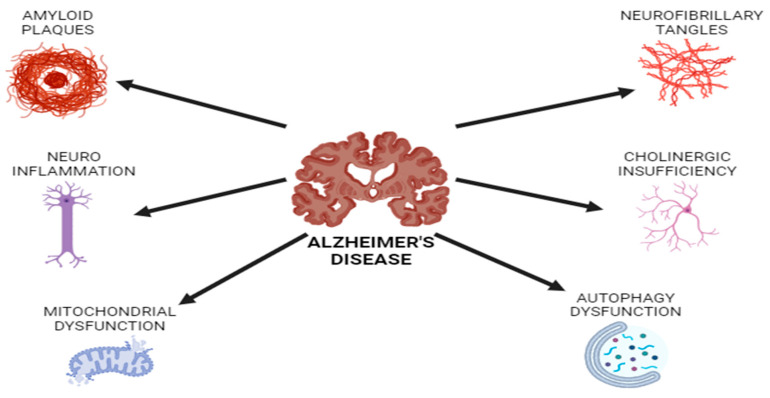
The biology of Alzheimer’s disease includes the basic biological processes and mechanisms that contribute to the disease’s appearance and advancement. The various factors that contribute to Alzheimer’s disease progression are shown in Figure 1. Amyloid plaques and hyperphosphorylated tau are the main factors that play a role. The development of senile plaques is facilitated by extracellular amyloid formation. The cytoskeleton and signal transmission in neural cells are disrupted by hyperphosphorylated tau, which also causes microtubules to break down. Neuroinflammation, oxidative stress, acetylcholine weakness, mitochondrial dysfunction, and autophagy failure all greatly contribute to the progression of the condition.

The primary etiological factor underlying dementia on a global scale is Alzheimer’s disease (AD). It is hypothesized that between 50 and 70 percent of all cases of dementia are attributable to this phenomenon. The projected tripling in dementia cases by 2050 represents a significant magnitude of the issue. At present, the total number of new cases reported annually exceeds 10 million [26]. It is estimated that 55 million people worldwide are currently affected by the disease, with indications that prevalence may quadruple in the near future, posing a significant public health challenge [27]. In its early stages, Alzheimer’s disease (AD) frequently presents without overt symptoms. The disease may progress at a gradual pace over an extended period of time. As a result, it can be challenging to implement effective treatment strategies and utilize appropriate early diagnosis techniques. It is not uncommon for individuals to be unaware that they are afflicted with Alzheimer’s disease (AD) until they begin to experience mild memory lapses [28]. The extent of the changes is amplified as the neurodegenerative processes in the brain progress, thereby marking the advent of more pronounced pathological alterations. In addition to other symptoms, patients may experience difficulties with activities of daily living, memory impairment, cognitive decline, mood swings, and sleep disturbances. As a consequence of the emergence of neurological and psychological symptoms, the individual affected by the disorder frequently requires assistance with activities that would otherwise be considered routine [29]. It is of significant importance to highlight that the mortality rate associated with Alzheimer’s disease (AD) has exhibited a marked increase, reaching 146.2% between the years 2000 and 2018. Currently, the disease is the sixth leading cause of mortality among older individuals in the United States, which illustrates the gravity of the situation [28]. As shown in Figure 3 the post-mortem examinations of brain tissue from individuals diagnosed with Alzheimer’s disease (AD) have revealed the presence of extensive accumulations of hyperphosphorylated tau protein (tau-p) and β-amyloid (βA). When the proteins coalesce, they form neurofibrillary tangles within the cells. A number of factors contribute to the progression of Alzheimer’s disease (AD). These include a sedentary lifestyle, smoking, excessive alcohol consumption, inadequate vitamin D intake, poor dietary habits, air pollution, lack of education, depression or chronic stress, diabetes, hypertension, dyslipidemia, obesity, cardiovascular disease, traumatic brain injury, elevated homocysteine levels, and oral and vascular diseases [30].

The ketogenic diet has potential therapeutic benefits for persons with Alzheimer’s disease (AD) [31]. An alternative term for the condition, often referred to as type 3 diabetes mellitus, highlights its biochemical and genetic characteristics, which in the future may be utilized to differentiate between type 1 and type 2 diabetes. Furthermore, hyperglycemia increases the risk of Alzheimer’s disease [32]. This notion is corroborated by a number of significant pieces of evidence, including the existence of brain insulin resistance in individuals with underlying health issues. There is a substantial body of evidence to support this claim [21]. The development of insulin resistance in AD patients can be attributed to alterations in the signaling pathways of the brain. The rising popularity of prepared foods that align with the characteristics of the Western diet can be attributed, at least in part, to societal changes [33]. Research conducted in 2022 provides definitive evidence of a causal relationship between this factor and the development of neurodegenerative processes, diabetes mellitus, Alzheimer’s disease, and insulin resistance in the brain. The primary glucose transporters in the brain are GLUT1 and GLUT3, which are also associated with Alzheimer’s disease (AD) [34]. It has been demonstrated that this intervention is effective in reducing the levels of the aforementioned substances. The specific symptoms of Alzheimer’s disease are clearly correlated with the presence of hyperphosphorylated tau protein and the quantity of neurofibrillary tangles in the brain [35]. Two common causes of abnormalities in brain activity are insulin resistance and a reduction in the number of glucose receptors in the brain. The capacity of neurons to obtain energy sources is significantly constrained by these factors. A well-designed, customized, and organized ketogenic diet provides the ability to impact several important factors [36]. In patients with Alzheimer’s disease (AD), ketone bodies can efficiently supply energy to the brain, thereby bypassing reliance on glucose [24]. The combination of the ketogenic diet with calorie reductions may prove an effective therapeutic method [37]. A reduction in insulin and glucose levels is likely to result in a corresponding reduction in insulin resistance. Insulin resistance is a primary etiological factor in Alzheimer’s disease-related memory impairment. There are multiple potential mechanisms through which the ketogenic diet may reduce insulin resistance [38]. The HOMA-IR number is frequently employed as a means of quantifying insulin resistance. A number of studies have demonstrated that following the ketogenic diet is associated with a significant reduction in the index value, providing compelling evidence of the diet’s impact on insulin resistance. Following a 12-week period, the research demonstrated a significant reduction in the HOMA-IR index value, with a 62.5% decrease observed (from an initial value of 3.73 to 1.4). Another study revealed that a four-week intervention resulted in a 45.9% reduction in the index, which is approximately half of its initial value [39]. The impact of the ketogenic diet on the aberrant brain energy metabolism associated with Alzheimer’s disease (AD) and the potential avenues for reducing the formation of amyloid plaques have emerged as a prominent area of investigation in recent years [40]. Previous animal studies have demonstrated that the ketogenic diet, when compared to a normal diet, results in a reduction in the formation of amyloid plaques in the hippocampal region, a decrease in microglial activity, and an enhancement in cognitive abilities such as learning and spatial memory [41]. A further study demonstrated that the diet resulted in a 25% reduction in amyloid plaques in animals with Alzheimer’s disease [42]. The ketogenic diet exerts a dual effect: it prevents problems with oxidative phosphorylation and alters the production of genes linked to neurodegenerative illnesses such as Alzheimer’s disease, particularly those involved in the metabolism of the hippocampus [42]. In Alzheimer’s disease (AD), mitochondrial dysfunction results in a progressive decline in energy production capabilities. The formation of amyloid plaques and the presence of inflammatory processes are the distinctive characteristics of this condition. Nevertheless, research has indicated that a ketogenic diet may initiate processes that regulate mitogenesis, with the potential to stimulate the formation of new mitochondria.

Furthermore, it has been demonstrated to alleviate neuroinflammation resulting from various factors, including an increase in the production of reactive oxygen species (ROS) due to mitochondrial damage [43]. The addition of medium-chain fatty acids (MCFAs) to the diet has been demonstrated to be an effective strategy in cases where empirical data indicate a potential benefit. Due to their capacity for rapid conversion into ketones by the body, fatty acids exhibit a high degree of ketogenic potential. Individuals with mild to severe Alzheimer’s disease who were administered 30 g of MCT tablets on a daily basis exhibited a notable enhancement in ketone uptake within the brain, accompanied by a general increase in brain energy consumption. The production of ketones from medium-chain triglycerides (MCTs) in the brain serves to compensate for the lack of glucose in individuals with Alzheimer’s disease (AD). The number of ketones present in the blood plasma is directly correlated with this adjustment [44]. For AD patients, MCT oil has demonstrated approximately 80% efficacy in maintaining or enhancing cognitive performance [45]. Also, studies have shown that adding medium-chain triglycerides (MCTs) to a person’s diet improves their working memory and other cognitive skills, both in persons with Alzheimer’s disease (AD) and those without dementia [46]. MCTs may increase mitochondrial activity, lessen the negative effects of amyloid on brain neurons, and reduce the total amount of the protein, according to research using an animal model. These findings are substantiated by additional studies [47]. Furthermore, the addition of MCT oil to a ketogenic diet appears to confer benefits, as it facilitates the maintenance of a heightened state of ketosis, even when the diet incorporates a modest increase in carbohydrate intake relative to a ketogenic diet that does not utilize MCT oil. Subsequent studies have demonstrated a correlation between elevated β-hydroxybutyrate levels in the blood and enhanced memory and cognitive abilities. These studies employed a double-blind methodology wherein neither the subjects nor the researchers were aware of the allocation of the active substance and the placebo. Research has demonstrated that exogenous hydroxybutyrate has the capacity to influence cognitive function [48]. The objective was to enhance happiness, daily functioning, and cognitive skills in individuals diagnosed with Alzheimer’s disease (AD) over a period of 20 months. A study published in 2021 investigated the impact of a ketogenic diet on individuals with a formal diagnosis of Alzheimer’s disease. This research constituted the inaugural randomized controlled trial conducted on patients who had been formally diagnosed with the disease. Given the low-fat composition of the diet, the study sought to ascertain whether there were any differences in impact when compared with a conventional diet. Addenbrooke’s Cognitive Examination III (ACE-III) scores of patients who followed the ketogenic diet demonstrated an increase of between 2.12 and 8.70 points. The same was observed with regard to the Alzheimer’s Disease Cooperative Study Activities of Daily Living Inventory (ADCS-ADL) and the Quality of Life in Alzheimer’s Disease (QOL-AD). The subjects demonstrated a mean increase of 3.12 to 5.01 points on both tests. Additionally, the research observed minor adverse effects concurrent with favorable alterations in cardiovascular risk factors [49]. It is noteworthy that 50% of the patients elected to continue the ketogenic diet beyond the 12-week trial period, despite their repeated assertion that they encountered difficulties adhering to its prescribed regimen. The notable enhancement in the quality of life for patients with Alzheimer’s disease may have a more pronounced influence than the various advantages of pharmaceuticals, such as cholinesterase inhibitors, on the disease. In light of the most recent scientific study results, it is imperative to subject the recommendations for low-fat diets for individuals with Alzheimer’s disease to rigorous scrutiny [50].

### 3.2. Parkinson’s Disease (PD)

Parkinson’s disease (PD) is a common neurological degenerative disorder characterized by central nervous system (CNS) involvement. Its incidence has doubled since 1990. It is particularly prevalent in the geriatric population, affecting 1% of people aged 60 years and older. However, it is becoming more common in the younger population. It will cause 5.8 million disability-adjusted life years worldwide in 2019, an increase of 81% since 2000. This trend is a major contributor to the global prevalence of disability. It affects an estimated 53 million people worldwide. It was responsible for 329,000 deaths in 2019, an increase of more than 100% since 2000. Manifestations of the disorder include impaired motor function, tremors, impaired balance, and atypical sensations or signs of neurological and psychiatric disorders. Dopamine synthesis and various physiological processes are significantly influenced by the substantia nigra, a region where neuron loss causes symptoms [51]. In these circumstances, the ketogenic diet may be beneficial, and its efficacy is the subject of extensive research.

**Figure 4:**
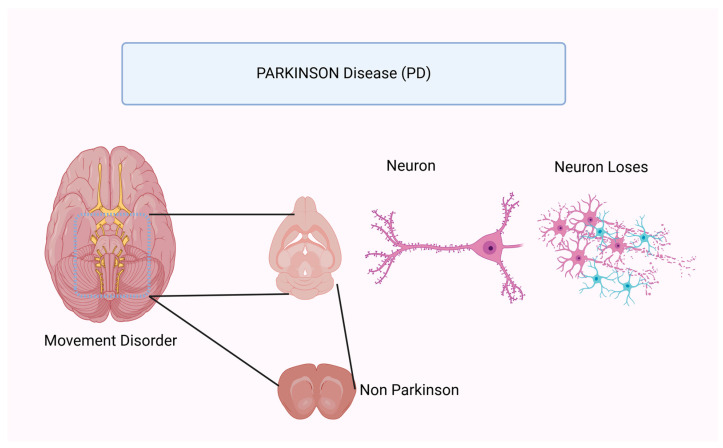
This new figure illustrates the key molecular pathways involved in Parkinson’s disease and how the ketogenic diet may influence them.

The Figure 4 shown that the ketogenic diet has the potential to influence Parkinson’s disease through several pathways that arise from the underlying characteristics of the disease. Numerous factors have been identified in relation to the disease, although the exact causes have not been definitively established. These elements include persistent inflammation in the nervous system, impaired mitochondrial function, an excess of reactive oxygen species (ROS), reduced dopamine synthesis, abnormal glucose metabolism in the brain, and the presence of damaged proteins known as Lewy bodies [52]. Research has shown that these parameters can be influenced by the ketogenic diet. The putative anti-inflammatory effects of the ketogenic diet on neurodegenerative diseases have been the subject of previous discourse. In this context, the diet exhibits a wide range of behaviors [53]. However, research has been conducted specifically to investigate the anti-inflammatory properties of the diet in relation to Parkinson’s disease. The anti-inflammatory effects of the ketogenic diet in PD are associated with modulation of the Akt/GSK-3/CREB signaling pathway, according to research published in 2022. Histones in the promoter region of the metabotropic glutamate receptor 5 (mGluR5) are acetylated to induce the desired modulation in a rat model of Parkinson’s disease [54]. Compared to the therapeutic use of a ketogenic diet, this study strongly supports the neuroprotective benefits of preventive ketosis in a rat model of lipopolysaccharide-induced Parkinson’s disease (LPS). After PD induction (by LPS), the model showed increased regulation of inflammatory mediators (TNF-α, IL-1 and IL-6), decreased dopaminergic neuronal expression, and decreased phosphorylation levels of the Akt/GSK-3/CREB pathway.

In addition, the model showed decreased mGluR5 mRNA, decreased mGluR5 acetylation in the mGluR5 promoter region, and decreased mGluR5+ microglial cell numbers [55]. These concerns were significantly reduced by the use of a preventive ketogenic diet. PET imaging was used to non-invasively identify and monitor the anti-inflammatory effects of the KD on Parkinson’s disease, which were precisely linked to histone acetylation or DNA methylation of the mGluR5 gene. The KD showed neuroprotective properties and reduced the inflammatory response associated with microglial activation. The ketogenic diet (KD) has been shown to reduce inflammation in PD by altering the mGluR5/Akt/GSK-3β/CREB signaling cascade. This alteration was achieved by increasing the level of histone acetylation in the promoter region of the mGluR5 gene. There is also speculation about the potential of a ketogenic diet (KD) to enhance the neuroprotective mechanisms responsible for attenuating neuroinflammation in Parkinson’s disease. Preventing the formation of the NLRP3 inflammasome, inhibiting the activation of pro-inflammatory genes, inducing epigenetic changes associated with calorie restriction, providing polyunsaturated fatty acids, reducing reactive oxygen species (ROS), and modulating the composition of the gut microbiome are some of these mechanisms at work. In addition to their role as an energy source, ketones also act as signaling molecules. DNA methylation and histone acetylation are some of the epigenetic mechanisms that can affect microglial cells. They may provide neuroprotection by regulating neuroinflammation [56]. Due to the strong relationship between increased reactive oxygen species (ROS), defective mitochondria, and abnormal glucose metabolism, the ketogenic diet may influence these factors. By promoting the synthesis of new mitochondria, ketone bodies can significantly enhance the production of ATP and mitochondrial respiration. The reduction in free radicals is due to the respiratory chain in mitochondria functioning more efficiently [57]. The main ketone body, beta-hydroxybutyrate, has the potential to reduce the symptoms of Parkinson’s disease, such as loss of dopaminergic neurons and mitochondrial insufficiency [58]. Given the critical consequences that can result from dopamine deficiency, the preservation of dopaminergic neurons is essential. L-DOPA, sometimes referred to as levodopa, is a well-established dopamine precursor that is widely used in the treatment of Parkinson’s disease. However, abnormal accumulation of α-synuclein, to which the drug may contribute, can increase the formation of Lewy bodies [59]. Recent research has shown that the combination of levodopa and a ketogenic diet is significantly more effective than using the drug alone [60]. Ketones have been shown to have a neuroprotective effect on dopaminergic neurons, preventing their degeneration in Parkinson’s disease models. When beta-hydroxybutyrate (BHB) was injected subcutaneously into rodents and administered to cortical neurons in a controlled laboratory environment, the observed phenomenon occurred.

In addition, ketone bodies have been found to improve motor function and reduce the number of pro-inflammatory cells in the brain [61]. The ketogenic diet may affect PD through an additional mechanism involving an indirect effect on the gastrointestinal microbiome. Diet is known to influence the composition of the gut microbiota. In addition, research has shown that the microbiome contributes significantly to the progression and development of PD [62]. A randomized controlled trial involving 47 patients diagnosed with Parkinson’s disease was conducted in 2018. Out of a total of 38 participants, the study included two different groups: one group followed the ketogenic diet, and the other group maintained a low-fat, high-carbohydrate diet. After eight weeks, both groups showed signs of improvement. However, the improvement was more pronounced in the group that followed the ketogenic diet. The results of Part I of the Unified Parkinson’s Disease Rating Scale (UPDRS) for the ketogenic diet group showed a significant improvement of 41%, with a reduction of 4.58 to 2.17 points from baseline. The low-fat diet group, on the other hand, showed only an 11% improvement, with a reduction of 0.99 to 3.63 points. While non-motor symptoms showed this characteristic, the reduction in motor symptoms was comparable and statistically significant in both groups. While the low-fat diet group experienced intermittent worsening of tremor and/or rigidity, the ketogenic diet group reported increased appetite [63]. Over the course of 12 weeks, the effects of a ketogenic diet on 16 people diagnosed with Parkinson’s disease were studied. The research also revealed the beneficial effects of the diet. A significant increase in the Parkinson’s Anxiety Scale was observed. At the same time, there was an increase in waist circumference, body mass index (BMI), triglycerides, glycated hemoglobin, fasting insulin, HDL cholesterol, and C-reactive protein (CRP). No significant changes were observed in symptoms of depression as measured by the CSD-R-20, the Centre for Epidemiological Studies’ depression scale. In a separate study, the introduction of a ketogenic diet in Parkinson’s disease patients was shown to result in an average reduction of 10.72 points on the Unified Parkinson’s Disease Rating Scale (UPDRS), a reduction of almost 50% from baseline. In a short period of 28 days, significant improvements were seen in body balance, walking technique, reduction of uncontrollable tremors, emotional state, and general vitality [64]. To compare the effects of a ketogenic diet with those of a high-carbohydrate diet, independent research was conducted on people with Parkinson’s disease. Although there were no noticeable differences in motor function between the two groups, as measured by the UPDRS, the group following the ketogenic diet showed improved cognitive abilities, particularly in terms of short-term memory and verbal fluency, compared to the other diet [65]. A case report of a 69-year-old woman with Parkinson’s disease and moderate symptoms of depression and anxiety was published in 2022. The study investigated the therapeutic use of the ketogenic diet. The individual’s depressive symptoms on the Centre for Epidemiologic Studies Depression Scale (CESDR) decreased by 8 points, from 42 to 34. There was also a reduction on the Parkinson Anxiety Scale (PAS) from 23 to 17, a significant improvement of six points. A reduction in the likelihood of developing cardiovascular disease was observed as a consequence of the substantial improvement in all health indicators. On the UPDRS, however, scores increased from 24 to 33 [66]. Finally, five PD patients followed a ketogenic diet of 90% fat, 8% protein, and 2% carbohydrates for 28 days; their UPDRS scores improved, and Wojarek referenced these findings in his 2019 publication. Another study looked at how the ketogenic diet affected voice quality, which is impaired in Parkinson’s patients. The researchers used the Vocal Handicap Index (VHI) tool to assess the voice quality characteristics of people with PD, and the results showed that these factors had improved, suggesting that a different type of medication may be able to help people with PD improve their voice quality. Given the significant effects of the ketogenic diet on multiple factors and the potential for future treatment advances in Parkinson’s disease, further studies in this area are urgently needed [67].

### 3.3. Huntington’s Disease

Epilepsy is a chronic neurological disorder characterized by frequent and recurrent seizures. The disease is generally associated with a low risk of death, and most patients have a good prognosis, as evidenced by the absence of seizures. However, it is associated with a significant decline in general health. Although the disease can affect people of any age and sex, it is more common in children, with a slightly higher prevalence in men. It is considered one of the most common neurological disorders in children. It affects around 50 million people worldwide. The prevalence of active epilepsy in the population is 6.38 per 1000 people. It mainly affects people living in low- and middle-income countries. Consequently, a significant proportion of the world’s population is affected. It is important to emphasize that not everyone who has seizures has epilepsy. Sudden damage to the central nervous system can also cause seizures [68]. Today, people with drug-resistant epilepsy are often given medication, but some epileptics do not benefit from these treatments. This represents about 33 percent of all epileptics, including 20–40% of adolescents and 30–40% of adults [69]. A ketogenic diet may be the only remedy in such cases, as shown by a thorough meta-analysis among other studies.

**Figure 5:**
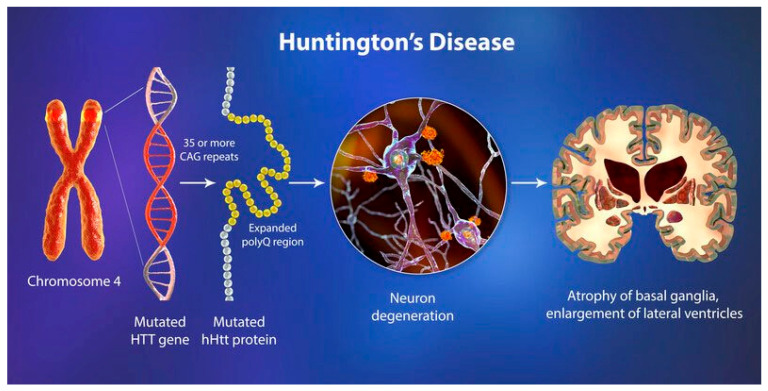
We have included a figure that highlights the neuroprotective effects of the ketogenic diet on Huntington’s disease pathology, focusing on mitochondrial dysfunction and neuroinflammation.

Although the ketogenic diet was the main therapeutic method for epilepsy a century ago, pharmacological treatments became the dominant technique after 1940. For example, the anti-epileptic drug Dilantin has largely replaced the ketogenic diet because of its efficacy and ease of use. Doctors are increasingly favoring this treatment as a therapeutic method due to its effectiveness in reducing epileptic seizures, which has led to a decline in the prescription of ketogenic diets [70]. As shown in Figure 5 the reason for this result is the ability to achieve similar results with less effort, as the use of the diet requires a more complete medical strategy than the prescription of drugs alone. As mentioned above, medication has been shown to be unsuccessful in about one-third of cases. Therefore, because of its efficacy in situations where conventional medicine fails, the ketogenic diet remains, after more than a century, a recognized therapeutic choice for drug-resistant epilepsy.

The ketogenic diet received renewed attention in 1997 with the release of the film *First Do No Harm*. The film chronicled the individual journey of Charlie Abrahams, the son of Jim Abrahams, a well-known Hollywood film producer. Charlie turned to the ketogenic diet as a last resort to control his epileptic seizures [70]. Existing research suggests that the ketogenic diet can effectively reduce epileptic seizures in a significant number of people, with reduction rates typically ranging from 50% to 90%. Around 27% of people have the potential for a significant 90% reduction in seizure frequency [71]. In 2022, an in-depth study was carried out on 160 pediatric patients, with an average age of five years and nine months, who were being treated for epilepsy using the ketogenic diet. The researchers monitored the effects of the diet over the course of 3, 6, 12, and 24 months. A percentage of children were found to have completely stopped having seizures, and this percentage varied depending on how long the seizures lasted: 13.7% after three months, 12.5% after six months, 14.4% after 12 months, and 10.6% after 24 months. On the other hand, 41.9% of children showed a reduction of at least 50% after three months, 37.5% after six months, 28.7% after 12 months, and 16.2% after 24 months [72]. A 2022 meta-analysis focusing on pediatric epilepsy reported that at least 50% of children experienced a reduction in seizure frequency, with 48.31% achieving complete seizure freedom. The analysis also found that children on the ketogenic diet were 5.6 times more likely to achieve a 50% or greater reduction in seizure frequency than those in the control group. A larger trial found that 35-56.1% of people with epilepsy on a ketogenic diet had a 50% or greater reduction in seizure frequency, compared with only 6-18% in the control group [73]. A meta-analysis was also carried out in 2020 to see how the ketogenic diet affected children with epilepsy under the age of two. The research included a collective of 534 individuals. The empirical data showed that up to 33% of the seizures stopped altogether, while 59% of the newborns experienced a significant reduction in seizure frequency of at least 50%. Based on the data obtained, scientists have determined that the ketogenic diet is both effective and safe for babies with drug-resistant epilepsy [74]. Currently, the modified Atkins diet (MAD) is increasingly being used as a replacement for the traditional ketogenic diet. The MAD is a variation of the ketogenic diet. A meta-analysis was conducted to evaluate the efficacy of the two dietary approaches. The classic ketogenic diet reduced seizure frequency by at least 50% in 62%, 60%, 52%, 42%, and 46% of patients after 1, 3, 6, 12, and 24 months, respectively. On the other hand, the modified Atkins diet reduced seizure frequency by 55%, 47%, 42%, and 29% after 1, 3, 6, and 12 months, respectively. The authors concluded that the efficacy of the two models was comparable. Another meta-analysis came to a similar conclusion. They found that there was a small advantage to the modified Atkins diet in terms of fewer adverse effects. In addition, the fact that the MAD is often more appealing may have an indirect effect on the overall effect by encouraging greater adherence to its requirements [75]. In addition, two other meta-analyses found similar results. Because of their potent ketogenic effects, MCTs are often recommended as an adjunct to the ketogenic diet. A single randomized controlled trial demonstrated the equivalent therapeutic efficacy of both dietary regimens. This meant that the average percentage of initial attacks was comparable at months 3, 6, and 12. The first trial of the diet showed 66.5% success after three months, 48.5% after six months, and 40.8% after one year. At 3, 6, and 12 months, the medium-chain triglyceride (MCT)-enriched version showed percentages of 68.9%, 67.6%, and 53.2%, respectively. Despite more than a century of research, the processes by which the ketogenic diet reduces the frequency of epileptic seizures are still not fully understood. There are recognizable features that may reduce the likelihood of seizures, although the exact mechanisms are unclear [44]. Numerous investigations have demonstrated the potential for ketones to act as anticonvulsants [76]. The year 2019 saw the completion of a study that added fresh insights into the molecular processes behind ketone bodies.

The level of β-hydroxybutyrate in the bloodstream is directly connected to how well the ketogenic diet prevents seizures. The potassium voltage-gated channel subfamily Q (KCNQ1/3) channels are stimulated by β-hydroxybutyrate, which helps to avoid seizures [76]. Furthermore, ketone bodies may exert an influence on brain metabolism, specifically with regard to mitochondrial activity and synaptic function, which may represent a potential mechanism of action. The hypothesis posits that glucose diffuses readily through the blood–brain barrier due to its high rate of absorption by neurons. This diffusion occurs within the brain capillary endothelium. Glucose is a necessary substrate for initiating neuronal seizure activity. The ketogenic diet is designed to reduce the frequency of seizures by limiting the availability of glucose and, therefore, energy, primarily by decreasing the rate at which neurons use glucose. This hypothesis is supported by findings indicating that hyperglycemia significantly affects the seizure threshold [77]. MCTs have been demonstrated to exert a range of effects and are frequently incorporated into ketogenic diets. In addition to elevating ketone levels within the body, ketones also enhance the concentration of decanoic acid in plasma. In comparison to ketones, the acid has been demonstrated to exert a more pronounced anticonvulsant effect due to its capacity to selectively inhibit the α-amino-3-hydroxy-5-methyl-4-isoxazolepropionic acid receptor (AMPA) [78]. An investigation carried out in 2020 verified the remarkable efficacy of medium-chain fatty acids. When MCT oil was given twice a day with meals for three months, adult patients with incurable epilepsy experienced a noteworthy 42% decrease in seizure frequency [71]. There is evidence of an increase in mitochondrial metabolism that occurs when the body is in ketosis, which increases the creation of ATP. This increase in ATP production causes the activation of ATP-sensitive potassium channels (KATPs), which in turn reduces the excitability of neurons. Studies have demonstrated that the ketone body acetoacetate (Acac) can block voltage-dependent Ca2+ channels (VDCCs) and reduce excitatory postsynaptic currents (EPSCs) in regions linked to epileptic activity. As such, it can effectively prevent convulsions in living organisms [35]. Ketones’ impact on improving the synthesis of the neurotransmitter GABA by lowering aspartate concentrations is thought to be another possible route. Consequently, a higher level of GABA might have an inhibitory effect on the start of convulsions. Furthermore, it has been suggested that ketones may increase the synthesis of the anticonvulsant neurotransmitter A1 adenosine and decrease glutamate levels, an excitatory neurotransmitter. Additional potential pathways encompass the regulation of intestinal microbiota, the mitigation of pro-inflammatory cytokines, the epigenetic impacts of β-hydroxybutyrate via the inhibition of class I histone deacetylases, and the influence on transcription of genes, specifically those linked to antioxidant components. These multifactorial effects are thought to contribute significantly to the diet’s efficacy in epilepsy management [72].

### 3.4. Multiple Sclerosis (MS)

The central nervous system (CNS), which encompasses the brain and spinal cord, represents the primary target of the autoimmune disease known as multiple sclerosis (MS). The disease is characterized by chronic inflammation and the degradation of nerve cells. The condition affects approximately 2.8 million people globally, comprising individuals of all genders and age groups. Furthermore, it is the primary cause of impairment among the younger population. It is regrettable to note that there has been an increase in the incidence of this condition, with 2.3 million individuals affected in 2013. Demyelination represents the pathological process that characterizes multiple sclerosis [79]. This condition results in the disruption of the conduction of nerve impulses, caused by damage to the myelin sheaths that surround and protect neurons. The disease may manifest in a number of ways, with variations observed between individual patients. The condition may manifest in either a progressive or a relapsing-remitting form. The most commonly reported symptoms include anxiety, fatigue, vertigo, visual impairments, aberrant sensations (dysesthesia), muscular exhaustion, and impaired balance [80]. The empirical evidence suggests that the ketogenic diet may confer certain benefits with regard to the treatment of MS. While ensuring appropriate use and safety, it may have an impact on the prevention and progression of the disease. The ketogenic diet has been proposed as a potential means of rebuilding and restoring myelin sheaths, given the evidence of demyelination processes observed in MS patients. In a 2022 publication, the authors posit that a Mediterranean-style ketogenic diet may prove more efficacious in influencing these processes [81]. The ketogenic diet has been demonstrated to affect brain-derived neurotrophic factor (BDNF), the principal growth factor produced by neurons and involved in myelin regeneration. The diet’s mechanism of action is enabled by β-hydroxybutyrate, a ketone molecule with the capacity to traverse the blood–brain barrier. Furthermore, it exerts an influence on mitochondrial respiration and NF-κB, which in turn promotes p300/EP300 histone acetyltransferase, thereby indirectly increasing BDNF synthesis. Furthermore, studies have demonstrated a negative correlation between blood glucose levels and BDNF expression [82]. The ketogenic diet has been demonstrated to affect brain-derived neurotrophic factor (BDNF), the principal growth factor produced by neurons and involved in myelin regeneration. The diet’s mechanism of action is enabled by β-hydroxybutyrate, a ketone molecule with the capacity to traverse the blood-brain barrier. Moreover, it exerts an influence on mitochondrial respiration and NF-κB, which in turn stimulates p300/EP300 histone acetyltransferase, thereby indirectly enhancing BDNF synthesis. Furthermore, studies have demonstrated a negative correlation between blood glucose levels and BDNF expression. The Mediterranean diet, which is rich in polyphenols, may also serve to amplify these effects by activating the CREB nuclear factor, thus enhancing its content and increasing the levels of BDNF [83]. Furthermore, 26 individuals diagnosed with multiple sclerosis participated in a separate study that examined the efficacy of the Mediterranean variation of the ketogenic diet. Following a four-month period of following the prescribed diet, significant improvements were observed in lean body mass, paraoxonase 1 (PON1), and ghrelin levels, while the sensation of fullness remained unchanged. The authors make a definitive assertion that the ketogenic diet based on the Mediterranean (isocaloric) model has a beneficial impact on the metabolic rate of their patients. A correlation has been identified between an enhanced perception of satiety and a diminished incidence of inflammatory conditions and oxidation processes. In light of the observed alterations in the parameters under investigation [84]. Dietary interventions exert an influence on the treatment of multiple sclerosis (MS) by modulating the concentration of the diagnostic marker for the disease, the serum neurofilament light chain (sNfL), which is associated with MS. This phenomenon was documented in a research investigation involving individuals who had received a diagnosis of relapsing-remitting multiple sclerosis. A reduction in sNfL levels was observed six months after the diet was initiated, which suggests that it exerts a neuroprotective effect in multiple sclerosis [85]. Further investigation revealed a significant anti-inflammatory effect, particularly among individuals with the ailment. A six-month randomized controlled trial involving 60 participants demonstrated this. In comparison to the control group, the ketogenic diet group exhibited a notable reduction in the expression of the arachidonate 5-lipoxygenase (ALOX5) gene. The synthesis of enzymes responsible for the production of pro-inflammatory eicosanoids is regulated by this gene. However, a notable decline in the synthesis of other enzymes that induce inflammation, particularly cyclooxygenase 1 (COX-1) and cyclooxygenase 2 (COX-2), was observed when comparing the outcomes of the same individuals before and after the implementation of dietary treatment. Moreover, a correlation was identified between the increased expression of pro-inflammatory genes and the reduced quality of life (MSQOL-54) in patients with multiple sclerosis [86]. A study conducted in 2022 involving 65 patients with recurrent MS reported notable improvements in neurological symptoms, mood disorders, and inflammatory markers after following a ketogenic diet for a period of six months. With regard to fatigue and despondency, the study participants exhibited a notable reduction of approximately 50%. With regard to both physical and mental health, improvement was demonstrated. As indicated by the Expanded Disability Status Scale (EDSS), the participants exhibited an improvement in their average disability status, with an average reduction in scores of 1.9 ± 1.1 points. Subsequent investigations into the potential advantages of MCT supplementation in conjunction with ketogenic diets for individuals with MS were unable to identify any statistically significant clinical improvements. Notwithstanding the aforementioned findings, there was a notable reduction in fasting glucose and insulin levels [87]. The ketogenic diet, which mimics fasting, includes additional processes that have been scientifically proven to exist, as demonstrated by another randomized controlled experiment. It has been demonstrated that a fast-mimicking diet stimulates the production of oligodendrocyte progenitors and facilitates the process of remyelination, which restores damaged axon function. It also lessens the symptoms of autoimmunization, which in turn lessens the symptoms of multiple sclerosis. Furthermore, T helper type 1 (TH1), T helper type 17 (TH17), and antigen-presenting cells (APCs) can lower their amounts. Additionally, research has demonstrated indications of possible benefits associated with the ketogenic diet for the treatment of relapsing-remitting multiple sclerosis (RRMS) patients [88].

### 3.5. Migraine

Migraine is the most prevalent neurological disorder. This condition has a global prevalence rate of 12%, with chronic migraine (CM) affecting between 1% and 2% of the global population. It is estimated that 2.5% of individuals initially diagnosed with episodic migraine will ultimately develop chronic migraine. Recurrent episodes of paroxysmal migraines may occasionally be severe. As a result, the quality of life and patients’ motivation to complete daily tasks are significantly diminished by this condition. Individuals who experience migraine are at an elevated risk of developing additional symptoms, including psychiatric disorders, sleep disturbances, and cardiovascular complications [89]. Recent clinical research has identified an expanding range of potential therapeutic benefits associated with the ketogenic diet for the management of migraines. The precise etiology of migraines remains unknown. However, it is hypothesized that dysfunctions in mitochondrial activity and issues associated with ATP synthesis may play a role in the development of the disorder. A diminished capacity to utilize glucose as an energy source may, on occasion, represent the underlying cause, which is a compromised metabolism of brain cells. It would appear, therefore, that ketone bodies represent a viable solution, in that they provide the brain with an alternative energy source. Nevertheless, it would be intriguing to consider the actual results of studies conducted on migraine sufferers in order to reach some fascinating conclusions. A randomized controlled trial was conducted to compare the effects of a strictly ketogenic diet with those of a non-ketogenic low-calorie diet. The study was conducted on a cohort of 35 individuals who met the dual criteria of having migraines and being overweight. The results demonstrated a mean reduction of 3.73 migraine days and 3.02 migraine episodes per month in those who adhered to the ketogenic diet, as opposed to those who followed the non-ketogenic diet. A minimum of 58% of patients in the ketogenic group experienced a reduction in the average number of migraine days by at least 50%. Conversely, the non-ketogenic group comprised a mere 8.57% of patients who attained an equivalent reduction [90]. In a further 2022 study comprising 23 individuals with a body mass index above the healthy range and a history of migraine, the ketogenic diet was found to result in a significant reduction in the mean number of days per month on which subjects experienced headaches, from 12.5 ± 9.5 days to 6.7 ± 8.6 days. Furthermore, the mean number of days on which medication was taken decreased from 11.06 ± 9.37 days to 4.93 ± 7.99 days, indicating substantial improvement. These changes were accompanied by reductions in body mass, adipose tissue, and BMI, indicating that the benefits extend beyond mere weight loss [8]. A distinct investigation examined 53 patients with treatment-resistant migraines and demonstrated the exclusive role of ketosis in achieving positive outcomes. Participants following the ketogenic diet showed significant reductions in the frequency and intensity of migraines and the need for medicines, while no similar improvements were observed in the low-carbohydrate group. These findings reinforce the notion that the therapeutic benefits of ketosis are driven by the process itself, rather than by carbohydrate restriction [27]. The impact of a dietary intervention on migraines unresponsive to pharmaceutical treatments was the subject of a distinct study. The dietary intervention implemented on patients suffering from drug-resistant migraine entailed the observance of a three-month ketogenic diet. Significant improvements were observed, as evidenced by a reduction in the average duration of pain from 30 to 7.5 days and in the average duration of pain episodes from 5.5 h. As determined by a pain rating scale, 83% of the participants reported experiencing the highest level of distress at the onset of the study. A reduction in the severity of distress was reported by 55% of the study participants after implementing the ketogenic diet. A 2022 meta-analysis comprehensively examined the ketogenic diet’s impact on migraines, demonstrating its capacity to significantly reduce attack frequency and severity [91].

The ketogenic diet (KD) has garnered significant interest for its potential benefits in treating neurodegenerative diseases.

## 4. Discussion

The ketogenic diet (KD) has demonstrated strong efficacy in the treatment of epilepsy, as supported by robust and extensive clinical evidence. However, its broader application in the management of other neurological disorders remains in the exploratory phase. Recent advancements in understanding the pathophysiology of neurodegenerative conditions such as Alzheimer’s disease, Parkinson’s disease, multiple sclerosis, and migraine have opened new avenues to investigate the therapeutic potential of KD in these areas. Although the current body of literature is growing, it remains limited compared to research focused on epilepsy. Our literature selection process was guided by specific inclusion and exclusion criteria. We prioritized peer-reviewed articles published between 2000 and 2024 that directly investigated the therapeutic effects or mechanisms of the ketogenic diet in neurodegenerative diseases. The studies included were primarily clinical trials, systematic reviews, meta-analyses, and mechanistic preclinical investigations. The studies excluded consisted of non-English publications, those lacking neurodegenerative relevance, or studies with insufficient methodological detail. Emerging studies also highlight the critical role of the gut–brain axis and the gut microbiota in neurodegeneration. The ketogenic diet has been shown to influence the composition of the gut microbiome, promoting the growth of anti-inflammatory microbial species. These changes may modulate central nervous system inflammation via microbial metabolites, such as short-chain fatty acids and neurotransmitter precursors, thereby contributing to improved cognitive and motor outcomes in neurodegenerative conditions. This gut–microbiota–brain interaction represents a novel mechanism through which KD may exert neuroprotective effects, particularly in diseases like Parkinson’s and Alzheimer’s. Another vital factor to consider is the impact of glycemic regulation on neurodegenerative disease progression. Elevated blood glucose levels, insulin resistance, and impaired cerebral glucose metabolism are well-documented contributors to neuronal dysfunction, especially in Alzheimer’s disease—often referred to as “Type 3 diabetes”. The ketogenic diet offers a therapeutic alternative by providing ketone bodies as a more efficient energy source for neurons, bypassing reliance on glucose. Additionally, ketone bodies have been shown to reduce insulin resistance and oxidative stress, thereby potentially mitigating disease severity. These metabolic advantages highlight the importance of glycemic control in preventing or slowing cognitive decline. While the therapeutic potential of KD is substantial, it is not without limitations. Gastrointestinal symptoms, including constipation, diarrhea, and vomiting, remain common side effects, particularly in pediatric epilepsy patients. In adults, adverse events such as kidney stones, menstrual irregularities, or fatigue have been observed but are generally infrequent. Notably, the transient symptoms known as “keto flu” are typically mild and self-limiting. Importantly, studies report no significant adverse effects of KD in patients with traumatic brain injury, and adverse reactions in Parkinson’s disease are usually mild and reversible. Despite these concerns, evidence to date suggests that KD modulates key pathways involved in neurodegeneration, including neuroinflammation, mitochondrial bioenergetics, and oxidative stress. These mechanisms may underlie the improvements in cognitive and motor function observed in early clinical studies across several disorders. However, a deeper molecular understanding of these effects is still required. While the ketogenic diet (KD) holds promising therapeutic potential for the management of neurodegenerative diseases, several limitations and challenges remain that must be addressed through future research. One of the key aspects that requires further investigation is the long-term efficacy and safety of the ketogenic diet. Current studies primarily focus on short-term clinical outcomes, with limited data on how sustained KD intervention affects the progression of neurodegenerative diseases over extended periods. Furthermore, the safety profile of KD, especially in populations with comorbid conditions such as cardiovascular disease, diabetes, and liver or kidney dysfunction, warrants thorough exploration to identify any long-term risks associated with its use.

Another critical area that remains under-explored is the specific mechanisms underlying the ketogenic diet’s effects on neurodegenerative diseases. While existing evidence supports the role of ketone bodies in reducing neuroinflammation, improving mitochondrial function, and protecting against oxidative stress, the precise molecular pathways and cellular targets through which these effects are mediated are not yet fully understood. For example, how KD modulates gene expression related to neurodegeneration, or interacts with the gut–brain axis, remains to be clarified. Further research should aim to uncover these underlying mechanisms to facilitate the design of more targeted, effective treatments.

Additionally, several practical challenges need to be addressed for the ketogenic diet to be widely adopted as a mainstream therapeutic strategy. These include issues related to dietary adherence, given the strict macronutrient ratios required for ketosis, and the potential for side effects such as gastrointestinal distress, weight loss, and metabolic imbalances. Personalized approaches to KD, taking into account genetic, metabolic, and disease-specific factors, could improve adherence and treatment outcomes. Moreover, the variability in individual responses to the ketogenic diet necessitates the development of tailored protocols to optimize its therapeutic potential for different patient populations.

In conclusion, while the ketogenic diet shows substantial promise as a therapeutic intervention for neurodegenerative diseases, further research into its long-term effects, safety, and underlying mechanisms is crucial. Overcoming the challenges of adherence and variability in response will be key to unlocking the full potential of this dietary intervention. The future of KD as a treatment for neurodegeneration lies in well-designed long-term clinical trials, more detailed mechanistic studies, and personalized approaches to therapy.

Future research should focus on long-term randomized controlled trials to confirm efficacy, understand inter-individual variability in response, and refine personalized KD protocols. Additionally, further investigation is needed into the interplay between metabolic shifts, gut microbiota composition, and neurodegeneration. Ultimately, through a personalized, systems-based approach, the ketogenic diet may emerge as a sustainable and effective therapeutic strategy for managing complex neurological conditions.

## 5. Conclusions

Significant global health challenges are presented by neurodegenerative diseases, such as Alzheimer’s, Parkinson’s, and Huntington’s disease. Promising results and a commitment to patient safety have established the ketogenic diet as an acknowledged therapeutic intervention for a variety of diseases. Myelin sheaths, which may be harmed in conditions such as multiple sclerosis, may benefit from the ketogenic diet, according to recent research. The brain-derived neurotrophic factor (BDNF), which is crucial for myelin regeneration in the brain, is influenced by the synthesis of ketone bodies like beta-hydroxybutyrate. In people with migraine, a common disorder, the ketogenic diet has shown promise in lowering both the frequency and severity of migraines. In contrast to those who adhere to non-ketogenic diets, studies have shown that individuals who strictly adhere to a ketogenic diet experience a reduction in the frequency and severity of migraines. It is essential to acknowledge the constraints and potential negative consequences associated with adhering to the ketogenic diet, despite its promising potential. Limited qualitative research on neurological disorders besides epilepsy constitutes a substantial limitation that restricts the quantity of information that can be obtained. Consequences such as gastrointestinal discomfort and metabolic irregularities may ensue if the diet is not appropriately modified and regulated. The ketogenic diet may, nevertheless, serve as a viable and secure therapeutic alternative for neurological disorders when administered with personalized care and adequate oversight. In conclusion, the ketogenic diet (KD) presents a promising therapeutic strategy for the management of neurodegenerative diseases, such as Alzheimer’s, Parkinson’s, and Huntington’s disease. Through its induction of ketosis and subsequent production of neuroprotective ketone bodies like beta-hydroxybutyrate (BHB), the KD has been shown to improve mitochondrial function, reduce oxidative stress, and modulate inflammatory pathways—critical factors in the pathophysiology of neurodegeneration. While its application in treating epilepsy is well established, emerging evidence supports the potential of the ketogenic diet to slow disease progression, enhance cognitive function, and alleviate motor symptoms in patients with various neurodegenerative disorders. Despite these encouraging findings, further research is needed to elucidate the precise mechanisms underlying the diet’s neuroprotective effects and to determine its long-term efficacy and safety in broader patient populations. Additionally, studies exploring the combination of the ketogenic diet with other therapeutic modalities could further enhance its therapeutic potential. This review underscores the need for a deeper understanding of dietary interventions in the context of neurodegenerative diseases and advocates for continued interdisciplinary research to fully unlock the benefits of the ketogenic diet in promoting brain health and slowing neurodegeneration.

## Figures and Tables

**Table 1:** Blood levels with a regular diet, ketogenic diet (with less than 20 g of carbs per day), and diabetic ketoacidosis [25].

Blood Level	Normal Diet	Ketogenic Diet	Diabetic Ketoacidosis
Glucose (mg/dL)	80–120	65–80	>300
Insulin (μU/L)	6–23	6.6–9.4	≈0
Ketone Bodies (mmol/L)	0.1	7–8	>25
pH	7.4	7.4	<7.3
Lactate (mmol/L)	0.5–1.0	0.5–1.5	>2.0
Free Fatty Acids (μmol/L)	400–500	800–1500	>2000
Triglycerides (mg/dL)	50–150	50–200	150–500

## Data Availability

The data supporting the findings of this review paper are derived from previously published studies and publicly available datasets. No new primary data were generated or analyzed during the course of this study. The data analyzed in this review are referenced in the citations provided within the manuscript. As this is a review article, no original datasets were created. All data used in the analysis of the therapeutic potential of the ketogenic diet in neurodegenerative pathophysiology were obtained from publicly available resources or studies cited throughout the paper.For further information, the datasets referenced can be found through the links provided in the respective references.
